# Usefulness of ^18^F‐fluorodeoxyglucose positron emission tomography/computed tomography for predicting the prognosis and treatment response of neoadjuvant therapy for pancreatic ductal adenocarcinoma

**DOI:** 10.1002/cam4.3044

**Published:** 2020-04-12

**Authors:** Takahiro Yokose, Minoru Kitago, Yohji Matsusaka, Yohei Masugi, Masahiro Shinoda, Hiroshi Yagi, Yuta Abe, Go Oshima, Shutaro Hori, Yutaka Endo, Kenji Toyama, Yu Iwabuchi, Ryo Takemura, Ryota Ishii, Tadaki Nakahara, Shigeo Okuda, Masahiro Jinzaki, Yuko Kitagawa

**Affiliations:** ^1^ Department of Surgery Keio University School of Medicine Tokyo Japan; ^2^ Department of Radiology Keio University School of Medicine Tokyo Japan; ^3^ Department of Pathology Keio University School of Medicine Tokyo Japan; ^4^ Biostatistics Unit, Clinical and Translational Research Center Keio University Hospital Tokyo Japan

**Keywords:** MTV, pancreatic ductal adenocarcinoma, PERCIST, PET/CT

## Abstract

**Background:**

The Response Evaluation Criteria in Solid Tumors (RECIST) for computed tomography (CT) is preoperatively used to evaluate therapeutic effects. However, it does not reflect the pathological treatment response (PTR) of pancreatic ductal adenocarcinoma (PDAC). The Positron Emission Tomography Response Criteria in Solid Tumors (PERCIST) for positron emission tomography (PET)/CT is effective in other cancers. This study aimed to confirm the usefulness of PERCIST and the prognostic utility of PET/CT for PDAC.

**Methods:**

Forty‐two consecutive patients with PDAC who underwent neoadjuvant therapy (NAT) and pancreatectomy at our institution between 2014 and 2018 were retrospectively analyzed. We evaluated the treatment response and prognostic significance of PET/CT parameters and other clinicopathological factors.

**Results:**

Twenty‐two patients who underwent PET/CT both before and after NAT with the same protocol were included. RECIST revealed stable disease and partial response in 20 and 2 cases, respectively. PERCIST revealed stable metabolic disease, partial metabolic response, and complete metabolic response in 8, 9, and 5 cases, respectively. The PTR was G3, G2, and G1 in 8, 12, and 2 cases, respectively. For comparing the concordance rates between PTR and each parameter, PERCIST (72.7% [16/22]) was significantly superior to RECIST (36.4% [8/22]) (*P* = .017). The area under the curve survival values of PET/CT parameters were 0.777 for metabolic tumor volume (MTV), 0.500 for maximum standardized uptake value, 0.554 for peak standardized uptake value corrected for lean body mass, and 0.634 for total lesion glycolysis. A 50% cut‐off value for the MTV reduction rate yielded the largest difference in survival between responders and nonresponders. On multivariate analysis, MTV reduction rates < 50% were independent predictors for relapse‐free survival (hazard ratio [HR], 3.92; *P* = .044) and overall survival (HR, 14.08; *P* = .023).

**Conclusions:**

PERCIST was more accurate in determining NAT’s therapeutic effects for PDAC than RECIST. MTV reduction rates were independent prognostic factors for PDAC.

## Graphical abstract

^18^F‐fluorodeoxyglucose positron emission tomography/computed tomography (PET/CT) was more accurate in determining the therapeutic effects of neoadjuvant therapy for pancreatic ductal adenocarcinoma than CT, which detects anatomical changes. The reduction rate of the PET/CT parameter was an independent prognostic factor for pancreatic ductal adenocarcinoma.

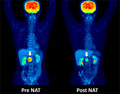

## 1 INTRODUCTION

Pancreatic ductal adenocarcinoma (PDAC) has a dismal prognosis,^1^ despite progress in diagnosis and treatment. PDAC treatment differs depending on whether the patient has a localized primary tumor or distant metastasis. Although surgical resection is the only potential curative treatment for PDAC patients without distant metastasis, the proportion of resectable PDAC at the time of diagnosis is approximately 20%‐30%.^2^ Local resectability is determined on radiography, and primary PDAC is defined based on the tumor extent considering vascular involvement as follows: potentially resectable PDAC (R‐PDAC), borderline resectable PDAC (BR‐PDAC), and unresectable PDAC (UR‐PDAC).^3^

Postoperative adjuvant chemotherapy is the standard treatment for patients with curative resected PDAC, as it improves prognosis.^4^ Furthermore, neoadjuvant therapy (NAT) increases the probability of R0 resection for UR‐PDAC and BR‐PDAC and performing curative resection improves prognosis.^5^ Among R‐PDAC patients, NAT decreased the recurrence rate and improved prognosis.^6^ Additionally, we reported the utility of neoadjuvant chemoradiotherapy (NACRT) for PDAC.^7^, ^8^, ^9^

The Response Evaluation Criteria in Solid Tumors (RECIST) guideline^10^ is followed for determining the anatomical target's tumor reduction ratio using CT images in order to evaluate treatment effect in many cancers. An abdominal contrast‐enhanced CT (CE‐CT) is widely used to evaluate the effect of NAT and prediction of resectability of PDAC.^11^ However, PDAC’s treatment effect is not evaluated appropriately using CT,^12^ and only 5% of studies used RECIST for PDAC.^13^

^18^F‐Fluorodeoxyglucose positron emission tomography (^18^F‐FDG‐PET) has advantages over CT because FDG uptake reflects tumor cell viability; moreover, FDG‐PET is superior to the RECIST for evaluating the treatment effect in various cancers.^14^, ^15^ The Positron Emission Tomography Response Criteria in Solid Tumors (PERCIST)^16^ using FDG‐PET to evaluate the treatment effect is useful for some cancers.^17^, ^18^ Some studies have evaluated the changes in the PET/CT parameters after NAT in PDAC,^19^, ^20^, ^21^ but only few studies have evaluated the therapeutic effect of NAT using PERCIST.^22^

The metabolic tumor volume (MTV) measured on FDG‐PET/CT reflects tumor cell activity and is associated with the prognosis of various cancers and locally advanced PDAC.^23^, ^24^, ^25^ In addition, MTV changes before and after NAT are prognostic factors in esophagus cancer,^26^ but to the best of our knowledge, they have not yet been reported as prognostic factors in PDAC patients who underwent NAT followed by surgery.

This study aimed to confirm the usefulness of PERCIST for evaluating the treatment effect based on the pathological treatment response (PTR) and to analyze the prognostic utility of FDG PET/CT parameters in PDAC patients who underwent NAT followed by surgery over a 5‐year period at our hospital.

## 2 MATERIALS AND METHODS

### 2.1 Patients

Sixty‐five consecutive patients underwent NAT for PDAC between January 2014 and October 2018 at Keio University Hospital. Inclusion criteria encompassed patients with histologically confirmed PDAC who underwent NAT followed by pancreatectomy. Among the 65 patients, 42 underwent pancreatectomy and were included in this study. Exclusion criteria removed patients who did not undergo FDG PET/CT before or after NAT using the same protocol. Among the 42 patients, 22 underwent both FDG PET/CT and CE‐CT before and after NAT using the same protocol. In all 22 patients, tumor resectability was evaluated using CE‐CT and FDG PET/CT approximately 2‐3 weeks before the initiation of NAT and approximately 2‐3 weeks after the completion of NAT, which included three cycles of neoadjuvant chemotherapy (NAC) or one cycle of NACRT. The patients underwent surgery approximately 30 days after NAT.

According to our institutional protocol, two NAT strategies were used: (a) the NACRT regimen comprising a combination of chemotherapy (concurrent TS‐1, cisplatin, and mitomycin) and radiotherapy (planned total dose, 40.0 Gy of external beam radiation therapy [40.0 Gy per 20 fractions])^7^, ^8^ and (b) NAC alone (gemcitabine and nab‐paclitaxel).

We conducted this retrospective observational study using the “opt‐out” method. The study was approved by the Human Experimentation Committee of our institution (No. 20120443), who waived the requirement for informed consent.

### 2.2 RECIST

CE‐CT images were retrospectively reviewed by two experienced radiologists (each with more than 8 years of experience in CT analysis) who were blinded to the other imaging results and clinical and histopathological data. Anatomical changes using CE‐CT were evaluated as described in RECIST version 1.1^10^ as follows: progressive disease (PD), >20% increase in tumor dimensions or the appearance of metastases; stable disease (SD), <30% shrinkage or <20% increase; partial response (PR), >30% decrease; and complete response (CR), complete disappearance of the primary target tumor. According to these categories, patients were considered responders (PR and CR) and nonresponders (PD and SD).

### 2.3 FDG PET/CT protocol

FDG PET/CT examinations were performed before the initiation of NAT and after the completion of NAT using the same protocol of the Biograph mCT system (Siemens Medical Solutions USA Inc). Quality assurance and quality control procedures for the PET system were carried out accurately on a daily basis. Patients were instructed to fast for 5 hours before the examinations. Blood glucose levels were measured immediately before the injection of FDG. None of the patients had a blood glucose level > 200 mg/dL. FDG was intravenously injected at a dose of 4.0 MBq/kg of body weight. PET scans were started after the uptake time (60 or 75 minutes), which was the same before and after NAT for each patient. Immediately after performing low‐dose plain CT for attenuation correction and anatomic localization, PET scans were obtained from the groin to the head for 120 seconds for each bed position in the three‐dimensional mode. The PET images were reconstructed using the three‐dimensional ordered subsets expectation maximization method with a 256 × 256 matrix, 3 iterations, 21 subsets, and a Gaussian filter of 6‐mm full width at half maximum.

### 2.4 PET analysis

FDG PET/CT images were retrospectively reviewed by two experienced nuclear medicine physicians (each with more than 8 years of experience in oncologic FDG PET/CT) who were blinded to the other imaging results and clinical as well as histopathological data. The commercially available software package GI‐PET (AZE Co., Ltd.) was used to objectively assess the treatment response based on PERCIST version 1.0.^16^, ^17^ The maximum standardized uptake value (SUVmax) was obtained, defined as the maximum single‐voxel intensity in the hypermetabolic lesion. Two parameters, the peak standardized uptake value corrected for lean body mass (SULpeak) and total lesion glycolysis (TLG), were used in PERCIST. The software automatically calculated these parameters within the entire primary tumor when a spherical region of interest (ROI) was drawn to encompass the primary lesion. In brief, the peak standardized uptake value (SUVpeak) was calculated in a 1.2‐cm‐diameter ROI placed on the hottest point of the tumor; the value was then normalized to the SULpeak (SUVpeak × [lean body mass]/[total body mass]) matched for Japanese individuals. The SULpeak was also used to determine whether that value for the tumor was > 1.5 times that of the mean liver SUL value + 2 standard deviations (SD); the mean liver SUL value was defined considering a 3‐cm‐diameter spherical ROI in the normal right lobe of the liver. MTV was defined as an FDG‐avid tumor volume with a threshold that is at least 1.5 times greater than the mean liver SUL value ± 2 SD. TLG was calculated as follows: SULmean × MTV (where SULmean represents the mean SUL value).

### 2.5 PERCIST

The PERCIST considers the percentage change in the SULpeak between the pre‐ and posttreatment scans^24^ as follows: progressive metabolic disease (PMD), >30% increase in the SULpeak; stable metabolic disease (SMD), <30% decrease or < 30% increase; partial metabolic response (PMR), >30% decrease; and complete metabolic response (CMR), complete disappearance of the SULpeak. According to these criteria, patients were considered responders (PMR and CMR) and nonresponders (PMD and SMD).

### 2.6 Pathological treatment response

The PTR was evaluated using hematoxylin and eosin staining of resected tumor sections; the findings were reviewed by an experienced pathologist who was blinded to the patient treatment and outcomes. Tumor cellularity, ie, the extent ratio of viable residual tumor cells at the primary tumor site, was assessed based on the estimated percentage of viable residual tumor cells compared with the tumor bed.

The PTR was classified into four categories based on the tumor cellularity, according to the College of American Pathologists,^27^ as follows: Grade 3, poor response defined as > 50% cellularity; Grade 2, moderate response defined as < 50% and > 10% cellularity; Grade 1, marked response defined as < 10% cellularity; and Grade 0, complete response defined as the complete disappearance of the tumor cells. According to these classifications, patients were considered responders (those with PR and CR as well as PMR and CMR with a Grade 0‐2; residual cellularity < 50%) and nonresponders (Grade 3).

### 2.7 Statistical analysis

Statistical analysis was performed using IBM SPSS statistics version 25.0 (IBM Japan, Tokyo, Japan). The paired *t* test was used to determine whether clinicopathological parameters were significantly different before and after NAT. The concordance rates and sensitivity were compared between PERCIST and RECIST using the McNemar test. The Pearson correlation coefficient was calculated. Paired *t* tests were used to compare the following:

(i) the absolute value of the difference between the tumor diameter change on CT scan before and after treatment and cellularity.

(i) = |post‐NAT tumor size/pre‐NAT tumor size × 100 (%) − cellularity (%)|

(ii) the absolute value of the difference between the SULpeak change rate on the PET/CT scan before and after NAT and cellularity.

(ii) = |post‐NAT SULpeak/pre‐NAT SULpeak × 100 (%) − cellularity (%)|

The optimal cut‐off values for continuous variables were estimated using receiver operating characteristic (ROC) curve analysis with the area under the curve (AUC). Relapse‐free survival (RFS) and overall survival (OS) were measured from the date of NAT initiation to the date of recurrence and the date of the last follow‐up, respectively. Kaplan–Meier curves for RFS and OS were generated, and comparisons between groups were performed using the log‐rank test. Variables with *P*‐values < .10 on univariate analysis were entered into a forward, stepwise backward multivariate Cox proportional hazards regression model to identify independent prognostic factors. Inter‐rater agreement of evaluation of treatment response between the two readers was analyzed using the Kappa coefficient. All statistical tests were two‐tailed, and *P*‐values < .05 were considered significant.

## 3 RESULTS

### 3.1 Patient characteristics

**Figure 1:**
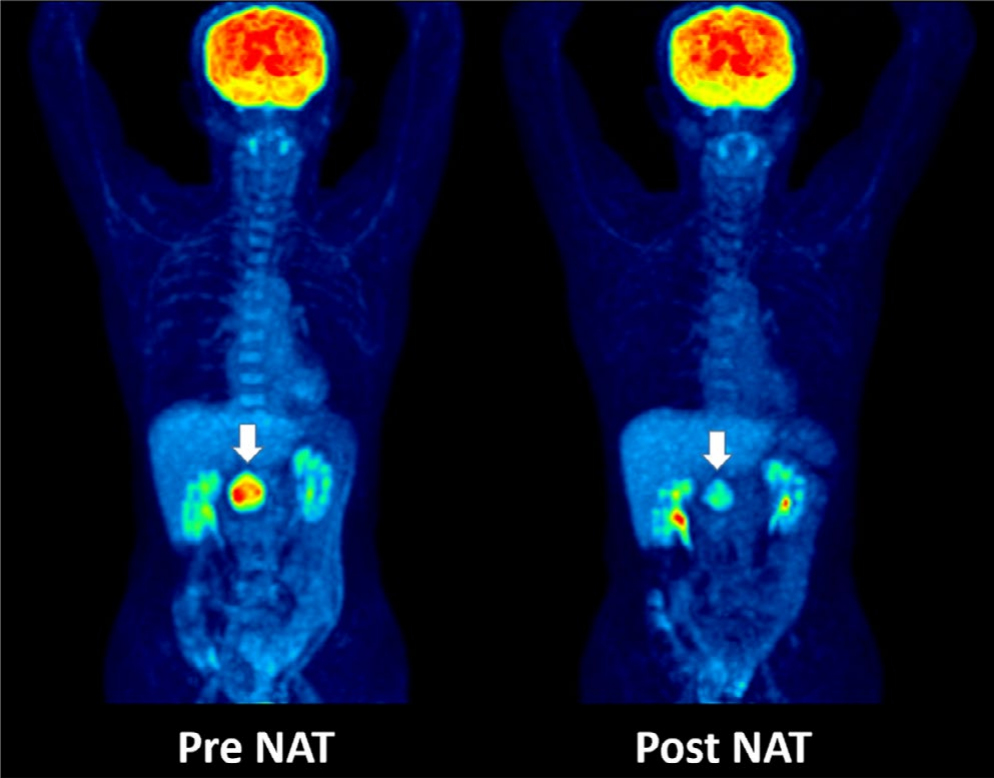
Representative FDG PET/CT examination before and after neoadjuvant therapy (NAT) revealed a significant decrease in FDG uptake in the tumor (arrow)

A total of 22 patients underwent PET/CT examination before and after NAT at our hospital (Figure 1). The patient characteristics are shown in Table S1. The median age was 70 years (range, 48‐78 years), and the male‐to‐female ratio was 17:5.

**Figure 2:**
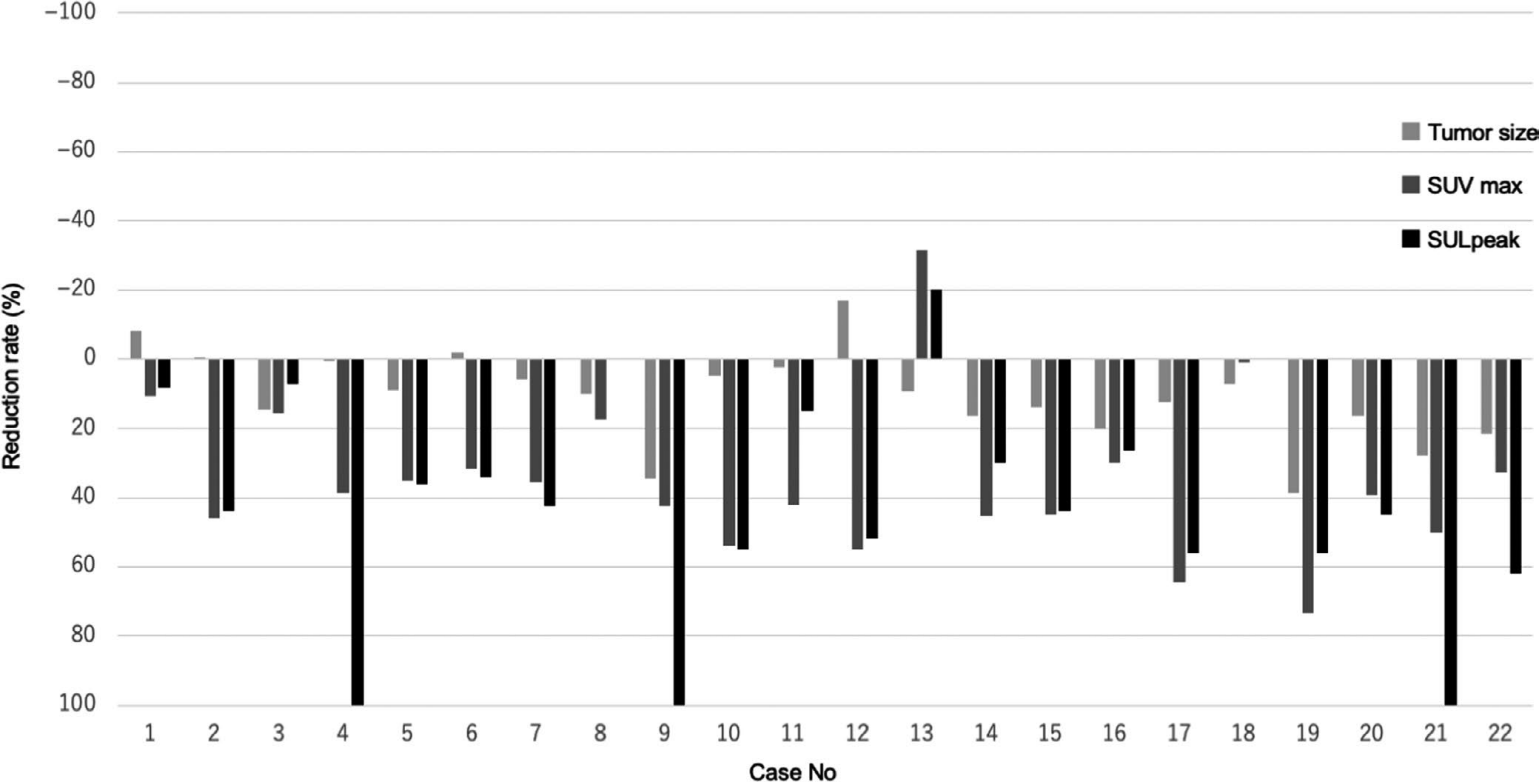
Waterfall plot analysis for the reduction rate of each parameter: tumor size, maximum standardized uptake value (SUVmax), and peak standardized uptake value corrected for lean body mass (SULpeak)

The tumor type was R‐PDAC, BR‐PDAC, and UR‐PDAC in 10, 11, and 1 case, respectively. As preoperative treatment, NACRT was performed in 18 and NAC in 4 cases. R0 resection was performed in 14 cases. The imaging parameters are shown in Table 1 and Figure 2.

**Table 1:** Imaging parameters pre‐ and post‐NAT and the reduction rate P‐values were estimated using the paired *t* test

Parameter	Pre‐NAT	Post‐NAT	*P*	Reduction rate	
	Mean ± SD			Median	Range
Tumor size	25.8 ± 6.7	22.7 ± 5.5	.002	9.6	−16.9‐38.6
SUVmax	6.42 ± 2.52	4.04 ± 2.05	<.001	38.8	−31.5‐73.3
SULpeak	4.18 ± 1.59	2.43 ± 1.51	<.001	43.2	−20.0‐100
MTV	14.66 ± 10.98	5.37 ± 6.62	0.001	65.2	−34.0‐100
TLG	48.04 ± 45.67	14.72 ± 21.11	0.001	64.7	−13.6‐100

Abbreviations: MTV, metabolic tumor volume; NAT, neoadjuvant therapy; SULpeak, peak standardized uptake value corrected for lean body mass; SUVmax, maximum standardized uptake value; TLG, total lesion glycolysis.

### 3.2 Comparing the prognostic utility of treatment response parameters

**Figure 3:**
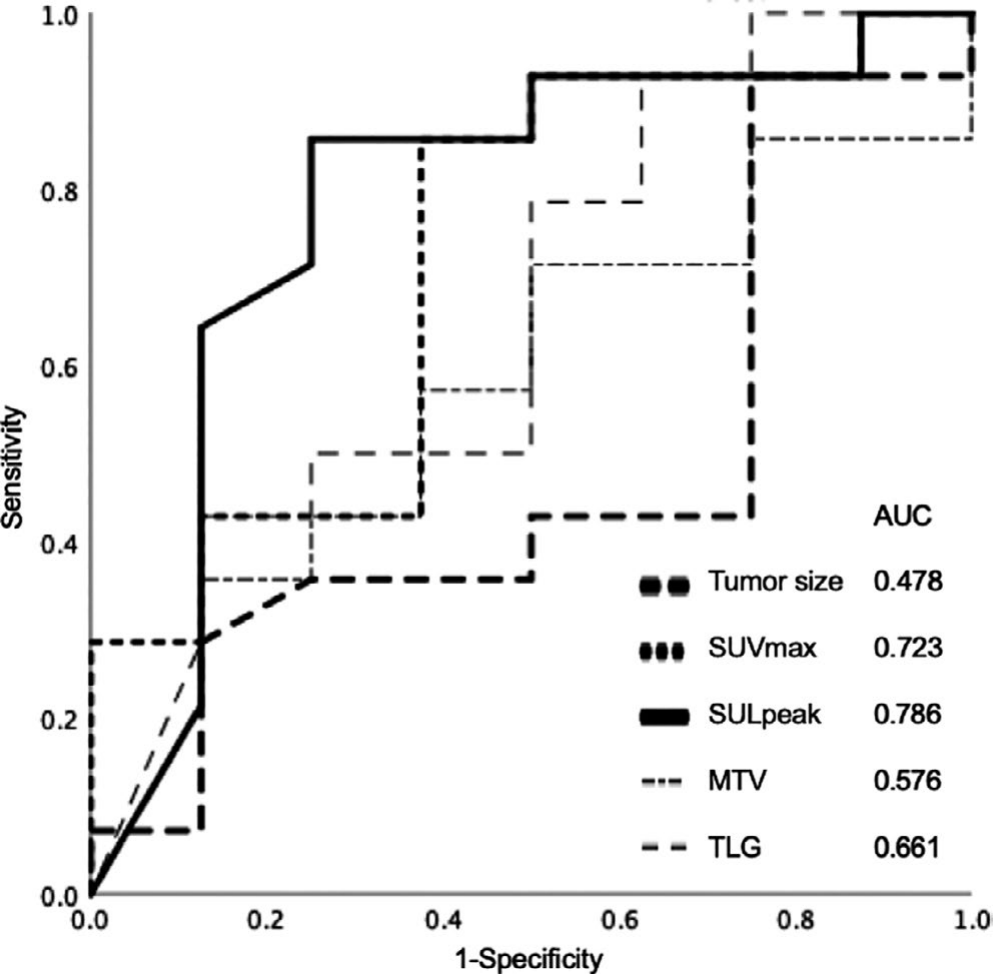
Analysis of receiver operating characteristic (ROC) curves to determine the best indicator for predicting the pathological treatment responders. The area under the ROC curve (AUC) values for the reduction rate of tumor size, maximum standardized uptake value (SUVmax), peak standardized uptake value corrected for lean body mass (SULpeak), metabolic tumor volume (MTV), and total lesion glycolysis (TLG) were 0.478, 0.723, 0.786, 0.576, and 0.661, respectively

To determine the best indicator for distinguishing responders from nonresponders, AUC was performed for the reduction rate of each parameter; the AUC values for tumor size, SUVmax, SULpeak, MTV, and TLG were 0.478, 0.723, 0.786, 0.576, and 0.661, respectively (Figure 3). Among the 22 patients, the treatment response according to RECIST was PR in 9.1% of patients (2/22) and SD in 90.9% (20/22). In contrast, according to PERCIST, 22.7% (5/22) of patients had CMR, 40.9% (9/22) had PMR, and 36.4% (8/22) had SMD. The PTR was G3 in 36.4% (8/22) of patients, G2 in 54.5% (12/22), and G1 in 9.1% (2/22) (Table 2). Inter‐rater agreements of evaluation of treatment response between the two readers were more consistent for PERCIST than RECIST; Kappa coefficients were 1.000 and 0.365, respectively.

**Table 2:** Treatment response according to the RECIST, PERCIST, and PTR. The patients were considered responders (CR and PR according to RECIST, CMR and PMR according to PERCIST, and G3 according to PTR) and nonresponders (PD and SD according to RECIST, PMD and SMD according to PERCIST, and G0‐2 according to PTR)

		PTR						Sensitivity, %	Specificity, %	Concordance rate, %
		Grade3	Grade2	Grade1		Grade0	Total			
		Nonresponders	Responders							
RECIST								7.1	87.5	36.4
PD	Nonresponders	0	0	0	0		0			
SD		7	12	1	0		20			
PR	Responders	1	0	1	0		2			
CR		0	0	0	0		0			
Total		8	12	2	0		22			
PERCIST								78.6	62.5	72.7
PMD	Nonresponders	0	0	0	0		0			
SMD		5	3	0	0		8			
PMR	Responders	2	7	0	0		9			
CMR		1	2	2	0		5			
Total		8	12	2	0		22			

Abbreviations: CMR, complete metabolic response; CR, complete response; PD, progressive disease; PERCIST, Positron Emission Tomography Response Criteria in Solid Tumors; PMD, progressive metabolic disease; PMR, partial metabolic response; PR, partial response; PTR, pathological treatment response; RECIST, Response Evaluation Criteria in Solid Tumors; SD, stable disease; SMD, stable metabolic disease.

For comparing the concordance rates between responders and nonresponders, PERCIST and PTR (72.7% [16/22]) were significantly superior to RECIST and PTR (36.4% [8/22]) (*P* = .017; Table S2). Furthermore, PERCIST was more sensitive for predicting cases with residual cellularity < 50%, observed in 78.6% of patients (11/14), than was RECIST (7.1% [1/14]) (*P* < .001; Table S2).

The change rate for the tumor size, SUVmax, and SULpeak revealed weak correlations with cellularity (*r* = .159, *r* = .454, and *r* = .426, respectively; Figure S1). On comparing the absolute difference between cellularity and the change rate for each parameter using the paired *t* test, SULpeak (35.96 ± 22.88 [95% confidence interval (CI), 25.81‐46.10]) and SUVmax (39.05 ± 23.62 [95% CI, 28.57‐49.52]) more accurately reflected cellularity than did RECIST (62.77 ± 26.71 [95% CI, 50.93‐74.61]; *P* < .001 and *P* = .001, respectively).

### 3.3 Survival analysis

At the time of analysis, 11 patients (50.0%) had recurrence and 8 (36.3%) had died. The median follow‐up from the date of NAT initiation was 35.5 months (range, 8.0‐54.4 months) for all 22 patients at the last follow‐up. The median OS and RFS were 39.0 months and 22.0 months, respectively.

**Figure 4:**
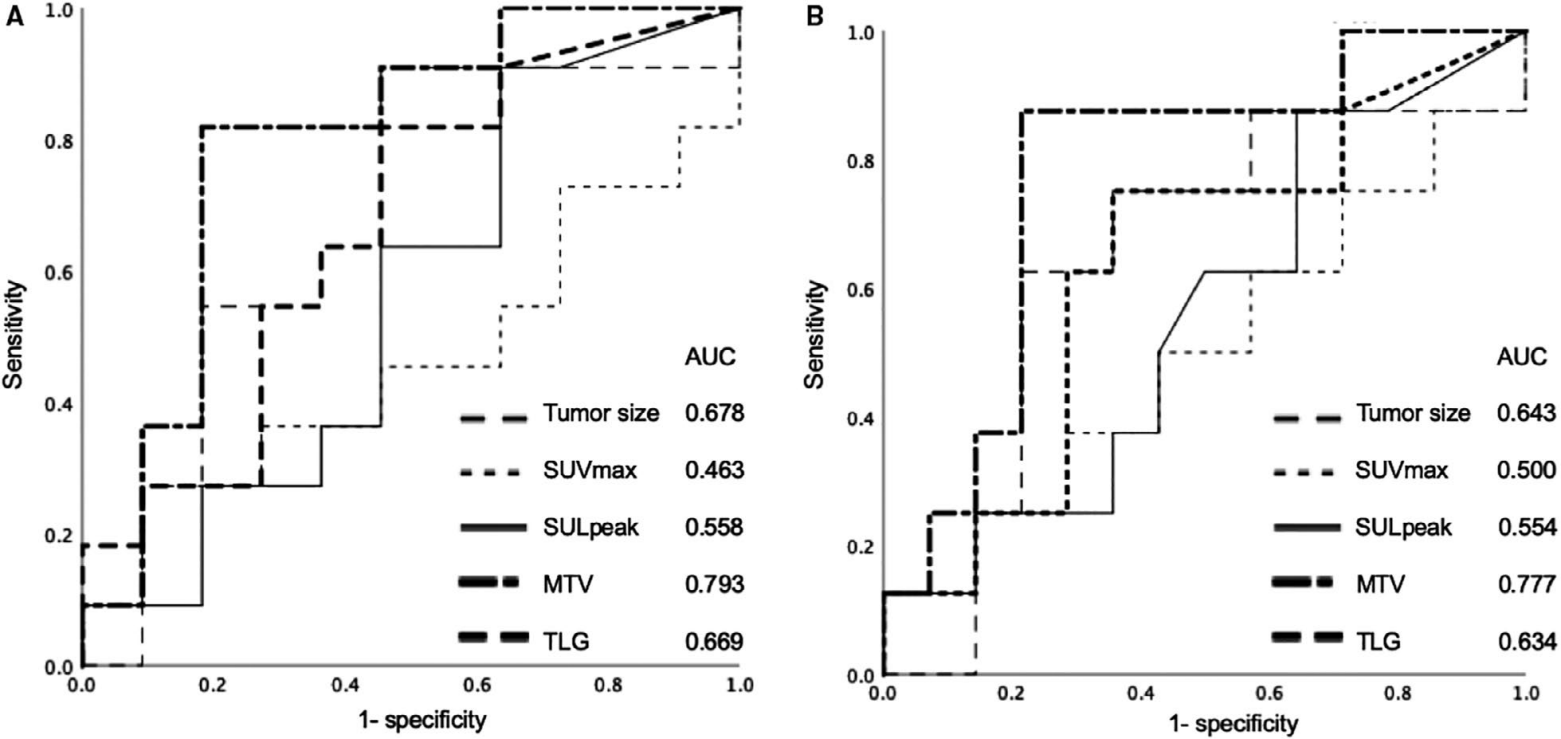
Analysis of receiver operating characteristic (ROC) curve to determine the best indicator for predicting recurrence (A) and prognosis (B). For recurrence, the area under the ROC curve (AUC) values for the reduction rate of tumor size, maximum standardized uptake value (SUVmax), peak standardized uptake value corrected for lean body mass (SULpeak), metabolic tumor volume (MTV), and total lesion glycolysis (TLG) were 0.678, 0.463, 0.558, 0.793, and 0.669, respectively. For survival, the AUC values for reduction rate of tumor size, SUVmax, SULpeak, MTV, and TLG were 0.643, 0.500, 0.554, 0.777, and 0.634, respectively

The cut‐off values for reduction rate changes in SUVmax, SULpeak, MTV, and TLG to recurrence and survival were examined using ROC curve analysis. MTV showed good predictive performance for recurrence and survival, consistently better than SUVmax, SULpeak, and TLG. The AUC recurrence and survival values were 0.793 and 0.777 for MTV, 0.463 and 0.500 for SUVmax, 0.558 and 0.554 for SULpeak, and 0.669 and 0.634 for TLG, respectively (Figure 4).

**Figure 5:**
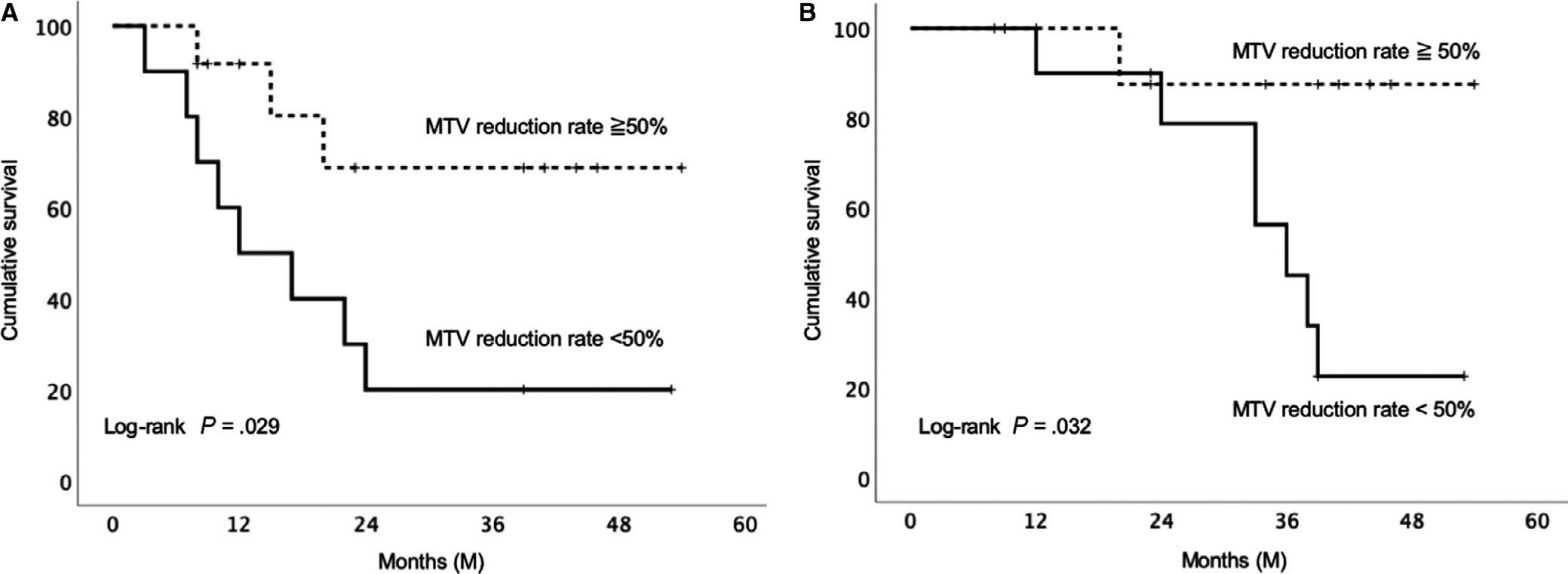
Survival curve using the Kaplan‐Meier method. A, The relapse‐free survival curve of patients with the metabolic tumor volume (MTV) reduction rate with a 50% cut‐off value. B, Overall survival curve of patients with the MTV reduction rate with a 50% cut‐off value

A 50% cut‐off value for the MTV reduction rate yielded the largest difference in RFS and OS between responders and nonresponders in the Cox regression analysis (hazard ratio [HR], 3.922, *P* = .044; and HR, 7.092, *P* = .068). Patients with > 50% reduction in MTV showed significantly longer 1‐year and 3‐year RFS (91.7% and 68.8% vs 50.0% and 20.0%, *P* = .029) and OS (100.0% and 87.5% vs 90.0% and 45.0%, *P* = .032) than patients with < 50% reduction in MTV (Figure 5). A significantly greater number of responders underwent NAC (*P* = .039) and longer NAT duration than nonresponders (*P* = .048). There was no significant difference in the other parameters between the groups (Table S3).

On univariate Cox regression analysis, sex and MTV reduction < 50% were significant predictors for RFS; tumor differentiation grade, tumor size, and MTV reduction < 50% were significant predictors for OS. On multivariate Cox regression analysis, MTV reduction < 50% (HR, 3.922 [95% CI, 1.040‐14.71]; *P* = .044) was an independent predictor for RFS (Table 3). Tumor differentiation grade (HR, 3.069 [95% CI, 1.37‐6.89]; *P* = .007) and MTV reduction < 50% (HR, 14.085 [95% CI, 1.44‐14.29]; *P* = .023) were independent predictors for OS (Table 3).

**Table 3:** Univariate and multivariate Cox regression analyses of prognostic factors for relapse‐free survival and overall survival

Variables	Relapse‐free survival					Overall survival				
	Univariate		Multivariate			Univariate		Multivariate		
	HR	*P*	HR	95% CI	*P*	HR	*P*	HR	95% CI	*P*
Gender	0.297	.090			.266	2.343	.246			
SUVmax reduction rate < 50%	1.319	.686				1.511	.618			
SULpeak reduction rate < 50%	1.818	.380				1.211	.817			
MTV reduction rate < 50%	3.922	.044	3.922	1.040‐14.71	.044	7.092	.068	14.08	1.439‐14.286	.023
TLG reduction rate < 50%	1.522	.490				1.473	.585			
Tumor differentiation grade	1.409	.219				2.105	.026	3.069	1.366‐6.894	.007
Pathological tumor size	0.694	.422				0.319	.089			.903
R1 resection	1.919	0284				2.889	.140			

Abbreviations: CI, confidence interval; HR, hazard ratio; MTV, metabolic tumor volume; SULpeak, peak standardized uptake value corrected for lean body mass; SUVmax, maximum standardized uptake value; TLG, total lesion glycolysis.

## 4 DISCUSSION

The results of this study showed that PERCIST and SUVmax were superior to RECIST for determining the therapeutic effect of PDAC preoperative treatment. Moreover, the MTV reduction rate of < 50% was an independent prognostic factor.

In this study, SULpeak was the best indicator for predicting patients who would become responders, considering the PTR and its accurate representation of cellularity. According to the RECIST, 1 of 2 patients with PR and 13 of 20 patients with SD were responders considering the PTR. PERCIST classified using SULpeak is superior to RECIST in determining the therapeutic effect of NAT on PDAC. The pancreas is a fibrous‐rich organ, which is better evaluated using ultrasonography,^28^ and PDAC is rich in fibrous components that are denatured and replaced by fibrous interstitial cells even if the number of actual tumor cells decreases after NACRT,^29^ which might correspond to poor changes in tumor size on CT scans. Accordingly, underestimating the therapeutic effect may result in delays in performing curative resection, thereby leading to cancer progression due to inefficient treatment.

In this study, the reduction rate of MTV before and after NAT was a better predictive parameter for recurrence and survival than other imaging parameters such as tumor size, SUVmax, SULpeak, and TLG according to ROC analysis. In addition, multivariate analysis showed that the MTV reduction rate was an independent prognostic factor for OS and RFS. Although there are some reports that PET parameters of the primary tumor or after NAT are associated with prognosis in advanced pancreatic cancer,^21^, ^25^ there are no reports analyzing the association between PET parameters and prognosis only in resected PDAC cases treated with NAT; our study is the first such report. MTV is an effective prognostic predictive parameter and a volume index that reflects the size and extent of tissue with high glucose metabolism. In fact, SUVmax is less reproducible owing to the susceptibility of statistical noise because of a single point value of glucose metabolism and does not reflect the tumor viability of the entire tumor.^16^ The discrepancy that MTV was useful for predicting prognosis but a poor factor for predicting PTR is probably because PTR is not necessarily a prognostic factor. In fact, <5% of residual tumor cells on pathological examination is reported as a good prognostic factor for PDAC,^30^ but cases with < 5% are very rare. In this study, one of two patients with G1, according to the PTR, experienced early recurrence, and PTR was not a prognostic factor. To predict the NAT therapeutic effect and prognosis on PDAC, evaluation of several FDG PET/CT parameters might be important.

This study had several limitations. First, we focused on cases treated with curative resection to investigate the correlation with histopathological treatment effects. According to our institution protocol, patients evaluated as having PD according to RECIST continued chemotherapy; therefore, they did not undergo curative resection or participate in the study. Accordingly, no patients had PD according to RECIST or PMD according to PERCIST in this study, but future studies are required for such patients. Second, this was a retrospective study with a heterogeneous study population. In addition, PERCIST can be used for evaluation only when PET/CT is performed using a consistent protocol. This criteria led to the exclusion of nearly half of the patients, which limited the sample size; thus, further prospective investigations in a larger number of cases are required. Finally, as PET/CT is expensive, this increases the medical costs, and not all patients might be able to undergo PET/CT before surgery.

In conclusion, PERCIST more accurately reflected NAT’s therapeutic effect on PDAC than RECIST. In addition, the change rate of MTV on PET/CT before and after NAT was an independent prognostic factor for PDAC. The precise evaluation of the PET/CT parameters may allow more appropriate prognostic stratification of PDAC, which leads to effective treatments for patients with PDAC.

## CONFLICT OF INTEREST

Masahiro Shinoda reports grants from Taiho Pharmaceutical Co., Ltd., Shionogi Co., Ltd., Eisai Co., Ltd., Novartis Pharma KK, Asahi KASEI Co., Ltd., Daiichi Sankyo Co., Ltd., outside the submitted work; Ryota Ishii reports personal fees from Kowa Company, Ltd., outside the submitted work; Shigeo Okuda reports grants from AZE Co., Ltd., outside the submitted work; Yuko Kitagawa reports grants from Taiho Pharmaceutical Co., Ltd., Chugai Pharmaceutical Co., Ltd., Yakult Honsha Co., Ltd., Daiichi Sankyo Co., Ltd., Merck Serono Co., Ltd., Asahi KASEI Co., Ltd., EA Pharma Co., Ltd., Otsuka Pharmaceutical Co., Ltd., Takeda Pharmaceutical Co., Ltd., Otsuka Pharmaceutical Factory Inc, Shionogi Co., Ltd., Kaken Pharmaceutical Co., LTD., Kowa Pharmaceutical Co., Ltd., Astellas Pharma Inc, Medicon Inc, Dainippon Sumitomo Pharma Co., Ltd., Taisho Toyama Pharmaceutical Co., Ltd., Kyouwa Hakkou Kirin Co., Ltd., Pfizer Japan Inc, Ono Pharmaceutical CO., LTD., Nihon Pharmaceutical CO., LTD., Japan Blood Products Organization, Medtronic Japan Co., Ltd., Sanofi KK, Eisai Co., Ltd., Tsumura Co., KCI Licensing, Inc, Abbott Japan CO., LTD., Fujifilm Toyama Chemical Co., Ltd., outside the submitted work. Other authors have nothing to disclose.

## AUTHOR CONTRIBUTIONS

Study concept and design: Yokose, Kitago, and Matsusaka. Analysis and interpretation of data: Yokose, Matsusaka, Masugi, Endo, Iwabuchi, Toyama, and Okuda. Drafting of the manuscript: Yokose. Critical revision of the manuscript for important intellectual content: Kitago, Matsusaka, Masugi, Shinoda, Yagi, Abe, Oshima, Hori, Nakahara, and Okuda. Statistical analysis: Takemura, and Ishii. Study supervision: Kitago, Jinzaki, and Kitagawa.

## Supporting information

FigS1

TableS1‐S3

Supinfo

## Data Availability

The data that support the findings of this study are available from the corresponding author upon reasonable request.
