# Supplementary figures and images for: Usefulness of 18F‐fluorodeoxyglucose positron emission tomography/computed tomography for predicting the prognosis and treatment response of neoadjuvant therapy for pancreatic ductal adenocarcinoma
Source: Cancer Med. 2020 Apr 12;9(12):4059–68. doi: 10.1002/cam4.3044 (PMC7300404; doi:10.1002/cam4.3044)

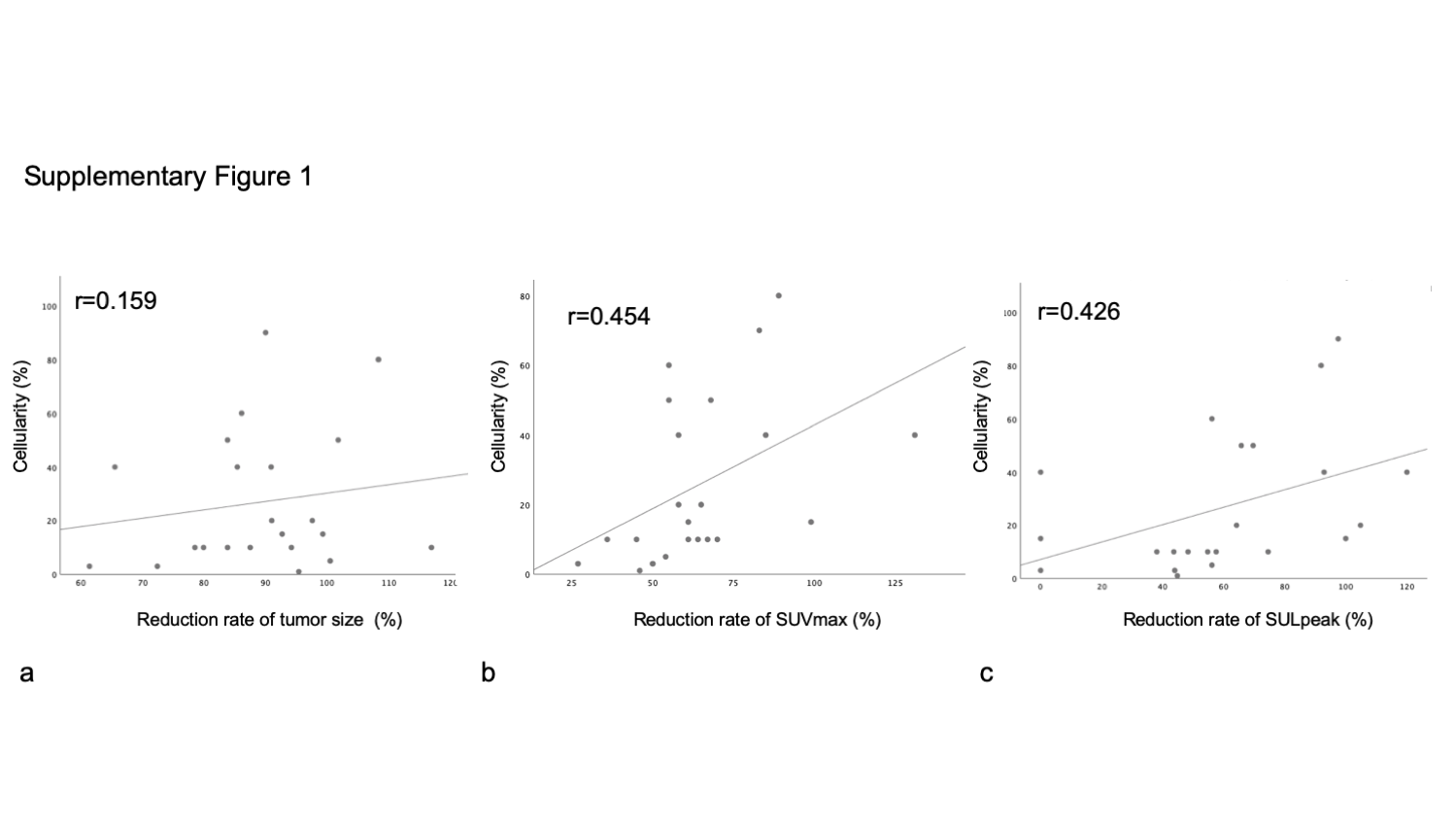

Supplement: Supplementary file 1 — FigS1 [file CAM4-9-4059-s001.tiff]
