# Supplementary material for: Usefulness of 18F‐fluorodeoxyglucose positron emission tomography/computed tomography for predicting the prognosis and treatment response of neoadjuvant therapy for pancreatic ductal adenocarcinoma
Source: Cancer Med. 2020 Apr 12;9(12):4059–68. doi: 10.1002/cam4.3044 (PMC7300404; doi:10.1002/cam4.3044)
Supplement: Supplementary file 3 — Supinfo [file CAM4-9-4059-s003.docx]

**Usefulness of ^18^F-Fluorodeoxyglucose Positron Emission Tomography/Computed Tomography for Predicting the Prognosis and Treatment Response of Neoadjuvant Therapy for Pancreatic Ductal Adenocarcinoma**

Yokose T, et al.

**4 Supporting information**

**Supplementary Table 1**. Patient characteristics

**Supplementary Table 2**. Comparison of the concordance rate and sensitivity between RECIST and PERCIST to predict PTR responders

**Supplementary Table 3**. Comparison of baseline characteristics and clinicopathological parameters according to whether the MTV reduction rate was less or more than 50%

**Supplementary Fig. 1**. Correlations between cellularity and each parameter are moderate: (A) tumor size according to Response Evaluation Criteria in Solid Tumors (RECIST), (B) maximum standardized uptake value (SUVmax), and (C) peak standardized uptake value corrected for lean body mass (SULpeak) according to Positron Emission Tomography Response Criteria in Solid Tumors (PERCIST).
